# *Lrp6* Genotype affects Individual Susceptibility to Nonalcoholic Fatty Liver Disease and Silibinin Therapeutic Response via Wnt/β-catenin-Cyp2e1 Signaling

**DOI:** 10.7150/ijbs.63732

**Published:** 2021-09-21

**Authors:** Li-Jie Chen, Xiu-Xian Lin, Jing Guo, Ying Xu, Song-Xia Zhang, Dan Chen, Qing Zhao, Jian Xiao, Guang-Hui Lian, Shi-Fang Peng, Dong Guo, Hong Yang, Yan Shu, Hong-Hao Zhou, Wei Zhang, Yao Chen

**Affiliations:** 1 Department of Clinical Pharmacology, Xiangya Hospital, Central South University, Changsha 410008, Hunan, China.; 2 Institute of Clinical Pharmacology, Central South University, Changsha 410078, Hunan, China.; 3 Engineering Research Center of Applied Technology of Pharmacogenomics, Ministry of Education, 110 Xiangya Road, Changsha 410078, P. R. China.; 4 National Clinical Research Center for Geriatric Disorders, 87 Xiangya Road, Changsha 410008, Hunan, P.R. China.; 5 Department of Pharmacy, Xiangya Hospital, Central South University, Changsha 410008, Hunan, China.; 6 Department of gastroenterology, Xiangya Hospital, Central South University, Changsha 410008, Hunan, China.; 7 Department of Hepatology and Infectious Diseases, Xiangya Hospital, Central South University, Changsha 410008, Hunan, China.; 8 Department of Pharmaceutical Sciences, School of Pharmacy, University of Maryland, Baltimore, Maryland 21201. USA.

**Keywords:** low-density lipoprotein receptor-related protein 6, nonalcoholic fatty liver disease, genotype, reactive oxygen species, cytochrome P450 2e1

## Abstract

**Background:** Nonalcoholic fatty liver disease (NAFLD) is a serious threat to human health worldwide, with a high genetic susceptibility. *Rs2302685,* a functional germline variant of *LRP6*, has been recently found to associate with NAFLD risk. This study was aimed to clarify the underlying mechanism associated with *rs2302685* risk and its impact on pharmacotherapy in treatment of NAFLD.

**Methods:** Venous blood samples were collected from NAFLD and non-NAFLD patients for SNP genotyping by using mass spectrometry. The *Lrp6*-floxdel mouse (*Lrp6*^(+/-)^) was generated to model the partial function associated with human *rs2302685*. The liver injury and therapeutic effects of silibinin were compared between *Lrp6*^(+/-)^ and *Lrp6*^(+/+)^ mice received a methionine-choline deficient (MCD) diet or normal diet. The effect of Lrp6 functional alteration on Wnt/β-catenin-Cyp2e1 signaling activities was evaluated by a series of cellular and molecular assays.

**Results:** The T allele of *LRP6 rs2302685* was confirmed to associate with a higher risk of NAFLD in human subjects. The carriers of *rs2302685* had reduced level of AST and ALT as compared with the noncarriers. The *Lrp6*^(+/-)^ mice exhibited a less severe liver injury induced by MCD but a reduced response to the treatment of silibinin in comparison to the *Lrp6*^(+/+)^ mice, suggesting Lrp6 as a target of silibinin. Wnt/β-catenin-Cyp2e1 signaling together with ROS generation could be exacerbated by the overexpression of Lrp6, while decreased in response to *Lrp6* siRNA or silibinin treatment under NAFLD modeling.

**Conclusions:** The *Lrp6* function affects individual susceptibility to NAFLD and the therapeutic effect of silibinin through the Wnt/β-catenin-Cyp2e1 signaling pathway. The present work has provided an underlying mechanism for human individual susceptibility to NAFLD associated with *Lrp6* polymorphisms as well as a rationale for the effective use of silibinin in NAFLD patients.

## Introduction

Nonalcoholic fatty liver disease (NAFLD) encompasses a spectrum of histologic injury to the liver, ranging from simple fat accumulation in hepatocytes, hepatic steatosis to necroinflammation or nonalcoholic steatohepatitis (NASH), in the absence of a history of significant alcohol use or other known liver diseases 1, 2. The prevalence of NAFLD has been increasing worldwide and is estimated to afflict approximately 25-30% of the general population, with variable outcomes ranging from a benign nonprogressive course, fibrosis, cirrhosis, decompensated liver disease, to hepatocellular carcinoma 3, 4. NAFLD not only affects the liver and gallbladder system, but also commonly associates with morbid obesity, diabetes, atherosclerosis, hyperlipidaemia, hypertension and metabolic syndromes with insulin resistance 5. Although certain environmental risk factors have been found to account for the development and progression of NAFLD, the diversity of the phenotypes in individuals with similar metabolic risk factors strongly implicates individual susceptibility to disease progression 6, 7.

In recent decades, familial, epidemiological and twin studies have suggested that genetic differences play a pivotal role in determining individual susceptibility to NAFLD 8, 9. Single nucleotide polymorphisms (SNPs) in the genes involved in hepatic lipogenesis, insulin resistance, inflammation, oxidative stress and fibrosis development have been associated with the severity of liver injury in NAFLD 10, 11. A number of candidate genetic variants have been reported to contribute to an individual's susceptibility to NAFLD, of which *rs738409 C>G*, which encodes the I148M protein variant of the patatin-like phospholipase domain-containing-3 (*PNPLA3*), has been identified as a strong determinant of liver fat accumulation that is not associated with body mass, dyslipidaemia and insulin resistance 3, 8, 9. However, only a small portion of the individual variabilities of NAFLD susceptibility could be explained by those reported candidate genetic variants. The knowledge of the genetic basis of NAFLD remains largely limited.

The 'two-hit hypothesis' is widely accepted in the etiology regarded as the mainstream pathogenesis of NAFLD. Here, the accumulation of lipids in the cytoplasm of liver cells (the first hit) triggers a series of cytotoxic events (the second hit), leading to an inflammatory response in the liver 7. During the second hit, oxidative stress plays a critical role in the pathogenesis of NAFLD. Oxidative stress leads to generation of reactive oxygen species (ROS), including superoxide radical, hydroxyl radical, hydrogen peroxide and lipid peroxide radicals, which cause inflammation and liver injury in the progression of NAFLD in a multistep process 12. It is increasingly believed that the genetic differences have a great impact on NAFLD outcomes through various inflammatory and stress signaling pathways 13, 14. Therefore, to explain the individual susceptibility to NAFLD, the SNPs in these pathways have been widely compared between NAFLD and non-NAFLD human subjects following the 'two-hit hypothesis'. Interestingly, the T allele of *rs2302685* in the gene (*LRP6*) encoding the low-density lipoprotein receptor-related protein 6 has been recently found to be more frequent in NAFLD patients, thus likely representing as a genetic factor of individual susceptibility to NAFLD 15-19.

Lrp6 is an important member of the low-density lipoprotein receptor family, serving as a key regulatory protein upstream of the Wnt/β-catenin signaling pathway 15. Usually, Lrp6 acts as a coreceptor with frizzled proteins for Wnt ligands to stimulate downstream signaling, which regulates β-catenin stability in the cytoplasm. The β-catenin can be translocated to the nucleus where it interacts with other transcription regulators, such as T-cell factors (TCF) and lymphoid enhancer factors (LEF), to affect the expression of key genes for cell proliferation, differentiation and tumorigenesis 20. Emerging evidence suggests that Lrp6 mutants can lead to dysregulation of the Wnt/β-catenin signaling pathway and subsequently metabolic syndromes. *R611C*, a rare nonconservative mutant of *Lrp6,* has been reported to be associated with autosomal dominant atherosclerosis that exhibits hyperlipidaemia and fatty liver disease 16. The *rs2302685*, a common functional germline variant of *LRP6*, has been reported to be associated with hyperlipidaemia and individual susceptibility to alcoholic liver disease (ALD) 17, 19. The cytochrome P4502E1 gene (*CYP2E1*) is one of the target genes regulated by the Wnt/β-catenin signaling pathway. Interestingly, oxidative stress usually exists in the liver of NAFLD patients with steatosis and particularly those with steatohepatitis, which is frequently associated with the induction of *CYP2E1*^21^. The knockout of *CTNNB1*, which encodes β-catenin protein, has been reported to result in the absence of *Cyp2e1* expression in mice 22. In contrast, abnormal activation of the Wnt/β-catenin signaling due to β-catenin mutations could increase the mRNA and protein levels of Cyp2e1 in liver tumour cells in mice 23. In addition, a decreased expression of *Cyp2e1* has also been reported in the *Lrp5/6* knockout mice 24. Because CYP2E1 is considered as one of the major regulators to on hepatic ROS levels during oxidative stress, the Lrp6-Wnt/β-catenin-CYP2E1 signaling axis might be an important mechanism accounting for individual susceptibility to NAFLD 19, 25.

While there are currently no effective medicines approved for NAFLD 26. Silibinin is considered to be one of the promising ones to treat the disease. Silibinin has considerably lower side effects as compared with other promising anti-NAFLD including antioxidant drugs (e.g., NOX-1/4 inhibitors), antifibrotic (e.g., galectin-3 antagonists, simtuzumab) and anti-inflammatory (e.g., sirtuins) agents 27, 28. Among NAFLD mouse models, the mice receiving methionine-choline deficient (MCD) diet are a stable, fast and widely used model in previous studies^10^, ^12^, ^29^, ^30^. With the hypothesis that the *Lrp6* genetic polymorphism influences individual susceptibility to NAFLD, the present work was aimed to ascertain whether the functional changes of Lrp6 caused by genetic mutation or knockdown have any impact on NAFLD progress and the therapeutic response to silibinin by using human clinical data, MCD diet-induced NAFLD mouse model, and cell models. The study might provide new targets for therapeutic development to prevent and treat NAFLD in the future.

## Methods and Materials

### Clinical patient blood sample and data collection

To discover germline variants contributing to individual susceptibility to NAFLD, we developed a protocol to collect blood samples from NAFLD patients and non-NAFLD controls. The study was performed in accordance with the principles of the Declaration of Helsinki and its appendices. The study was approved by the Medical Ethics Committee of the Department of Clinical Pharmacology, Xiangya Hospital, Central South University (Changsha, China) and registered with the Chinese Clinical Trial Registry (No. ChiCTR-ROC-15006899). All participants were provided with a written informed consent before participation. The NAFLD and non-NAFLD human subjects were recruited from the Health Management Centre of Xiangya Hospital, Central South University (Hunan, China) from June 2016 to February 2017. The diagnosis of NAFLD was made by ultrasonic imaging based on the guidelines for the diagnosis and treatment of NAFLD in China (2010), European Association for the Study of the Liver (EASL) and American Association for the Study of Liver Diseases (AASLD). Volunteers who were diagnosed with NAFLD but accompanied with other hepatic abnormalities were excluded from the NAFLD group. In the non-NAFLD group, the participants with a risk of NAFLD were also excluded. The patients with viral hepatitis, drug-induced hepatitis or alcoholic liver injury were excluded. Participants' clinical and demographic data, including age, gender, drinking history and biochemical laboratory measurements, were also recorded. The weight and height of the subjects were measured with a calibrated scale. The waist perimeter was determined as the midpoint between the lower border of the rib cage and the iliac crest. The hip circumference was measured at the widest circle of the bottom. Venous blood samples were obtained after a 12-hour overnight fast to determine fasting blood glucose (FBG), total cholesterol (TC), triglyceride (TG), high-density lipoprotein (HDL) and low-density lipoprotein (LDL), total bilirubin (TBIL), alanine aminotransferase (ALT) and aspartate aminotransferase (AST). All laboratory biochemical parameters were measured using a conventional automated analyser.

Venous blood samples (3-5 mL) were collected from each subject for DNA extraction using disposable venous blood lancets and disposable blood collection tubes. The genomic DNA was isolated using a commercial DNA extraction kit (Omega Bio-Tek, GA, USA) and used for SNP genotyping. We focused on the SNPs of the genes related to the 'two-hit hypothesis' that contribute to hepatic fat accumulation and oxidative stress response, as well as those SNPs distributed in the Wnt/β-catenin signaling pathway, apoptosis signaling, HIF signaling, NFKB signaling and JAK-STAT signaling pathways. The SNPs were mainly selected from the ENCODE database. All of the selected SNPs were missense variants with a Minor Allele Frequency (MAF) of > 0.05 in Han Chinese in Beijing and Han Chinese South according to the 1,000 Genomes Project. SNP genotyping was conducted by using mass spectrometry (Sequenom MassARRAY, XiangYa Medical Laboratory, Central South University, Changsha, China).

### Animal studies

The animal studies with mice were approved by the Ethics Committee of the Institute of Clinical Pharmacology, Central South University (Changsha, Hunan, China). All the animal handling and procedures complied with the relevant regulations of the Department of Experimental Animals, Central South University (Changsha, Hunan, China). A loxP-floxed Lrp6 C57/BL6 mouse line was generated in collaboration with Biocytogen (Beijing, China). This mouse line was crossed with a C57/BL6 Cre line (Biocytogen, Beijing, China), resulting in *Lrp6*-floxdel mice whose loxP-floxed exon 2 of the Lrp6 gene was completely deleted. The homozygotes of the Lrp6-floxdel mice (*Lrp6*^(-/-)^) usually died during embryonic development, but the homozygotes (*Lrp6*^(+/-)^) grew normally. However, the *Lrp6*^(+/-)^ mice have a reduced expression of Lrp6 and β-catenin, as well as the targeted genes of the Wnt/β-catenin pathway, such as Cyp2e1, as described in our recent study 19. The *Lrp6*^(+/-)^ mice were further employed in the present study.

The mice were housed in the Department of Laboratory Animals, Central South University (Changsha, China), in a specific pathogen free (SPF) animal centre. The centre temperature was controlled to 22 ± 2 °C, with the relative humidity set to 60 ± 5%. Days and nights were alternated in 12 h cycles. Animals had free access to water and food. Eight-week-old male mice were bred with the female mice of the same age. The pups were weaned after 21 days and numbered by ear tags. About 2 mm of each mouse's tail was cut off and placed in a 1.5 mL Eppendorf tube. DNA was extracted using a commercial mouse tail DNA extraction mini kit (Bimake.cn, Houston, USA). The *Lrp6* genotyping was performed according to the same method reported in our recent study 19.

For the subsequent experiments, the heterozygous* Lrp6*^(+/-)^ mice (*n* = 24) and the wide-type *Lrp6*^(+/+ )^ mice (*n* = 24) aged 8 weeks were randomly and evenly divided into three groups (n=8/each group) and treated as the following: NAFLD model groups without treatment (Group A) were fed a MCD diet; NAFLD model receiving silibinin treatment (Group B) were intragastrically administrated with 100mg/kg/d silibinin under the MCD diet; and the control groups (Group C) were fed a methionine and cystine supplement (MCS) diet. Sodium carboxymethyl cellulose (CMC-Na, 0.1mL/10g) was intragastrically administered in the NAFLD model and control groups as the vehicle control which was used to dissolve silibinin in the treatment groups. The status of the mice was observed and recorded every day during the experiment, and the mice were weighed once a week. After five weeks of treatment, the mice were euthanised. The blood samples were collected using eyeball extraction and then sent for biochemical analysis. Liver tissues were collected and stored in a -80 °C refrigerator (Thermo Scientific, MA, USA) for later histology, molecular and biochemical analyses.

### Cell culture and treatment

Cell experiments were further conducted to explore the possible mechanisms underlying the outcome differences of silibinin treatment in the animal experiments. HL7702 cell (kindly provided by the Central Laboratory, Xiangya Hospital, Central South University) were cultured in RPMI-1640 medium with 10% (*v/v*) foetal calf serum (GIBCO, Suzhou, China), 2.0 mM L-glutamine, 1.5 g/L NaHCO_3_, 0.1 mM nonessential amino acids, 1.0 mM sodium pyruvate, 100 IU/mL penicillin and 100 μg/ mL streptomycin. Cells were maintained in a 37 °C incubator (ThermoFisher, Mariette Ohio, USA) with 5% CO_2_ in humidified air.

The cell experiments were carried out as the following. Firstly, we investigated the effect of silibinin treatment on the absorption of oleate. HL7702 cells were plated in 12-well plates for 24 h and randomly assigned to the blank control, solvent control and 100 μM silibinin-treated groups in the presence of 200 mM oleate for another 24 h. Secondly, we determined whether the *Lrp6* knockdown by siRNA could affect the effect of silibinin treatment on oleate-induced cell damage.HL7702 cells were plated in 12-well plates for 8 h. After the cells reached more than 50% of the confluence, the plates were randomly assigned to blank control, vehicle control and *Lrp6* siRNA-treated groups. Cells in the *Lrp6* siRNA and vehicle control groups were transfected with *Lrp6* siRNA (75 ng) or empty vectors, respectively, using Lipofectamine™ RNAiMAX Transfection Reagent according to the manufacturer's protocol. After treatment for 24 h, the plates received silibinin or vehicle treatment in the absence or presence of oleate as described above. Thirdly, the impact of Lrp6 overexpression on the effect of silibinin treatment on oleate-induced cell damage was studied. HL7702 cells were plated in 12-well plates for 8 h. After more than 50% of their confluence, the plates were randomly assigned to Lrp6 overexpression, vehicle control and blank control groups. The cells in the Lrp6 overexpression and vehicle groups were transfected with the plasmid containing human wild-type (W) *Lrp6* (Tagged human wild-type *Lrp6* ORF clone was purchased from OriGene Technologies, Inc., Rockville, MD, USA) or that containing a *Lrp6* mutant (M) (The *Lrp6* [NM_002336] 3187 A>G mutant was constructed by Changsha Best Biotechnology Co., Ltd. Changsha, Hunan, China) with Lipofectamine 2000 (Invitrogen, ThermoFisher Scientific, USA) according to the manufacturer's protocol. After treatment for 24 h, the plates received silibinin or vehicle treatment in the absence or presence of oleate as described above.

### Reporter gene assays

To study how the functional status of Lrp6 affects the effect of silibinin treatment on the Wnt/β-catenin signaling activity, the TOPFlash HEK293 cells (University of Maryland, Baltimore, MA, USA) stably expressing a Wnt/β-catenin signaling reporter (the TCF/LEF were inserted into the upstream of a luciferase reporter gene) were used^31^. First, whether silibinin treatment could affect Wnt/β-catenin signaling was explored. The TOPFlash HEK293 cells (2×10^4^) were seeded in 12-well plates. Eight hours later, the plates were randomly assigned to the normal Dulbecco's Modified Eagle Medium (DMEM) (control), the Wnt3a-conditioned DMEM and the DMEM containing 25 mM LiCl, respectively. After 12 h, the medium in plates was changed to DMEM containing a solvent (vehicle control), 100 μM silibinin or 10 μM XAV939 (positive control) for another 12 h of cultivation. The cells were then lysed by 1×RIPA Lysis Buffer and harvested into 1.5 mL Eppendorf tubes using a scraper. After centrifugation at a speed of 20,379 g for 5 min at 4 °C, the supernatant was transferred for dual luciferase reporter gene assays that were carried out by referring to the manufacturer's protocol for the dual-luciferase reporter assay system kit (Promega Corporation, Wisconsin, USA). The luminescence of luciferase was measured on a luminometer (LUMIstar Omega, BMG LABTECH, Cary, USA). The data were analysed by the built-in software of the luminometer.

We also investigated the impact of Lrp6 expression on silibinin's effct on the Wnt/β-catenin signaling activity. The TOPFlash HEK293 cells (2 x 10^4^) were seeded in 12-well plates. Eight hours later, the cells were transfected with plasmids containing *Lrp6*W,* Lrp6*M or empty vector by using Lipofectamine 2000 (Invitrogen, ThermoFisher Scientific, USA) according to the manufacturer's protocol. Twenty-four hours later, the cells were treated with 100 μM silibinin or the solvent control for 12 h. The cells were lysed and the supernatant was used for dual luciferase reporter gene assays as described above.

### Biochemical measurement with mouse plasma

The plasma was separated from the collected mouse blood and sent to the Clinical Laboratory of Xiangya Hospital (Hunan, China) for the detection of ALT, AST and LDL-C plasma levels by using a fully automatic biochemical analyser (AU5821, Beckman, USA).

### Detection of Malondialdehyde (MDA), TG and TC in mouse liver tissues

Mouse liver tissue samples were accurately weighed and added to saline by a proportion of 1 g/10mL. The tissues were homogenated mechanically in an ice water bath and then centrifuged at 3135 g for 10 min at 4 °C. The protein concentration was determined using a bicinchoninic acid (BCA) assay kit (Beyotime Institute of Biotechnology, Shanghai, China). To measure the levels of MDA, TG and TC in the tissues, the samples were placed in a boiling water bath for 40 min, cooled down, and then centrifuged for 10 min at a speed of 5486 g at room temperature. The supernatants were transferred into a 96-well plate for determination of MDA, TG or TC by spectrophotometer using assay kits (Institute of Nanjing Jiancheng Biological Engineering, Nanjing, China). The determination wavelengths were 532 nm and 510 nm for MDA and TG/TC, respectively. The concentrations of MDA and TG/TC were calculated from the following formula: Content in tissues=optical density (OD) value in test tube - OD value in blank tube/(OD value in standard tube - OD value in blank tube)×standard substance concentration.

### H & E staining

Small pieces of mouse liver tissues were cut and submerged in 4% paraformaldehyde, embedded in paraffin, and sectioned into 5 μm thick slices. The slices were initially stained with haematoxylin (H) for 5 min and then washed in deionisation water. Eosin (E) staining was performed for 5 min, and the slices were rinsed again in deionisation water. Finally, the slides were dehydrated, cleared, and mounted. Images were taken with a Nikon ECLIPSE Ts2R inverted microscope (Nikon, Tokyo, Japan).

### Immunoblotting

The protein expressions of Lrp6, β-catenin and Cyp2e1 in mouse liver tissues and cells were determined by Western blot (WB) analysis. The total proteins from mouse liver tissues or cells were prepared by using RIPA (Beyotime Institute of Biotechnology, Shanghai, China), and the protein concentration was determined by using a BCA protein assay reagent kit (Beyotime Institute of Biotechnology, Shanghai, China). Protein samples were separated using 8% SDS-polyacrylamide gels before being transferred to polyvinylidene fluoride membranes (Millipore, Bedford, USA). After incubation with primary antibodies, the blot was then incubated with horseradish peroxidase (HRP)-conjugated secondary antibodies (Beyotime Institute of Biotechnology, Shanghai, China) before being imaged using an Immobilon Chemiluminesent HRP substrate and a ChemiDoc TM XRS^+^ Gel Imaging System (BIO-RAD, CA, USA). The band densities were analysed using Image Studio Lite (v. 5.2) software. The relative expression of the target protein was reported as the percentage of the OD of the target protein normalized with the corresponding OD of β-actin or GAPDH. The experiments were conducted in triplicates for each antibody. Western blot was carried out using antibodies specific for the proteins as followed: Lrp6 (sc-25317) and β-catenin (sc-7963) were purchased from Santa Cruz Biotechnology. Cyp2e1 (abcam), β-actin (abcam) and GAPDH (abcam) were purchased from Cell Signaling.

### Mouse microsomal Cyp2e1 activity

Mouse liver microsomes were prepared by using differential centrifugation as described previously 25. The microsomal activity of Cyp2e1 was evaluated by chlorzoxazone 6-hydroxylation. A 0.2 ml incubation mixture that consisted of the probe drug chlorzoxazone, liver microsomes (0.5 mg/ml) and 0.1 M sodium phosphate buffer (pH 7.4) was prewarmed for 5 min at 37 °C. The control incubation mixture was similar to the experimental groups, except that the liver microsomes were excluded. The reaction was initiated by the addition of 1 mg/mL of Nicotinamide Adenine Dinucleotide Phosphate (NADPH). The reaction incubations were performed in a shaking water bath (37 °C) for 30 min and stopped by adding 0.2 ml ice-cold acetonitrile, which contained glycyrrhetinic acid (23.3 ng/mL) as the internal standard (IS). The samples were injected into the UPLC-MS/MS system (A TRIPLE QUADTM 6500 UPLC-MS/MS system, AB SCIEX, Concord, Ontario, Canada) for analysis. The mass spectrometer was operated in negative ion mode using multiple reaction monitoring (MRM). The precursor-product ion transitions were monitored at m/z 132-168 for chlorzoxazone (CZX), m/z 120.1-184.1 for 6-hydroxy chlorzoxazone (6-OH-CZX) and m/z 469.3-355.3 for the IS. Data acquisition was performed using Analyst software version 1.4.2 (AB Sciex, Concord, Ontario, Canada). To determine the activity of Cyp2e1, the area ratio of 6-OH-CZX to its parent CZX was used. All reactions were carried out in triplicates.

### Detection of cellular

#### Lipid accumulation by Oil Red O staining

An Oil Red O staining kit (Nanjing Jiancheng Bioengineering Institute, Nanjing, China) was used according to the manufacturer's protocol. The Oil Red O dye was extracted from the cells and quantitated by a microplate reader (Thermo, France) at 490 nm.

#### Visualization of cellular lipids by inverted laser confocal microscope

For the visual observation of the changes in lipid content, the slides were placed in 12-well plates before cell seeding. After various experimental treatments, the slides were dipped in 4% formalin to fix the cells. The cell nucleus was stained with 1% DAPI solution, and 1 μM Nile red used to stain cellular lipids. The slides were then rinsed with PBS, mounted and allowed to dry. The lipid staining was examined with an inverted laser confocal microscope (Confocal A1-R1 microscope, Nikon, Japan) using a 552/636nm (excitation/emission) lamp setting.

#### Determination of cellular ROS levels

To determine the levels of ROS, the cells were incubated with 2.7-Dichlorodihydrofluorescein diacetate (10 mM) at 37 °C for 30 min. The reaction mixture was then aspirated and replaced with 200 µL PBS in each well. The plate was kept on a shaker for 10 min at room temperature in the dark. An inverted fluorescent microscope (Nikon ECLIPSE Ti-S, Japan) was used to visualise the intracellular fluorescence of the cells and capture the images.

### Statistical analysis

Statistical analysis was done using SPSS (IBM, v. 20.0) software. For the genotype data, the Hardy-Weinberg test was performed to calculate allelic frequencies using an χ^2^ test. The categorical variables were expressed as a percentage and assessed using a Student's t-test or χ^2^ test. A logistic regression analysis was performed to estimate the odds ratio (OR) and 95% CI for the association between the polymorphism and NAFLD with adjustment of the confounders (including age, sex, BMI). The data from cellular and animal experiments were expressed as the mean ± standard deviation (SD). The statistical analyses were performed using ANOVA test. A chi-square test was applied for nonparametric statistics, and significant differences were analysed using two-tailed tests. A *P* value < 0.05 was considered as statistical significant.

## Results

### *LRP6 rs2302685* was associated with reduced liver injury in NAFLD patients

**Figure 1:**
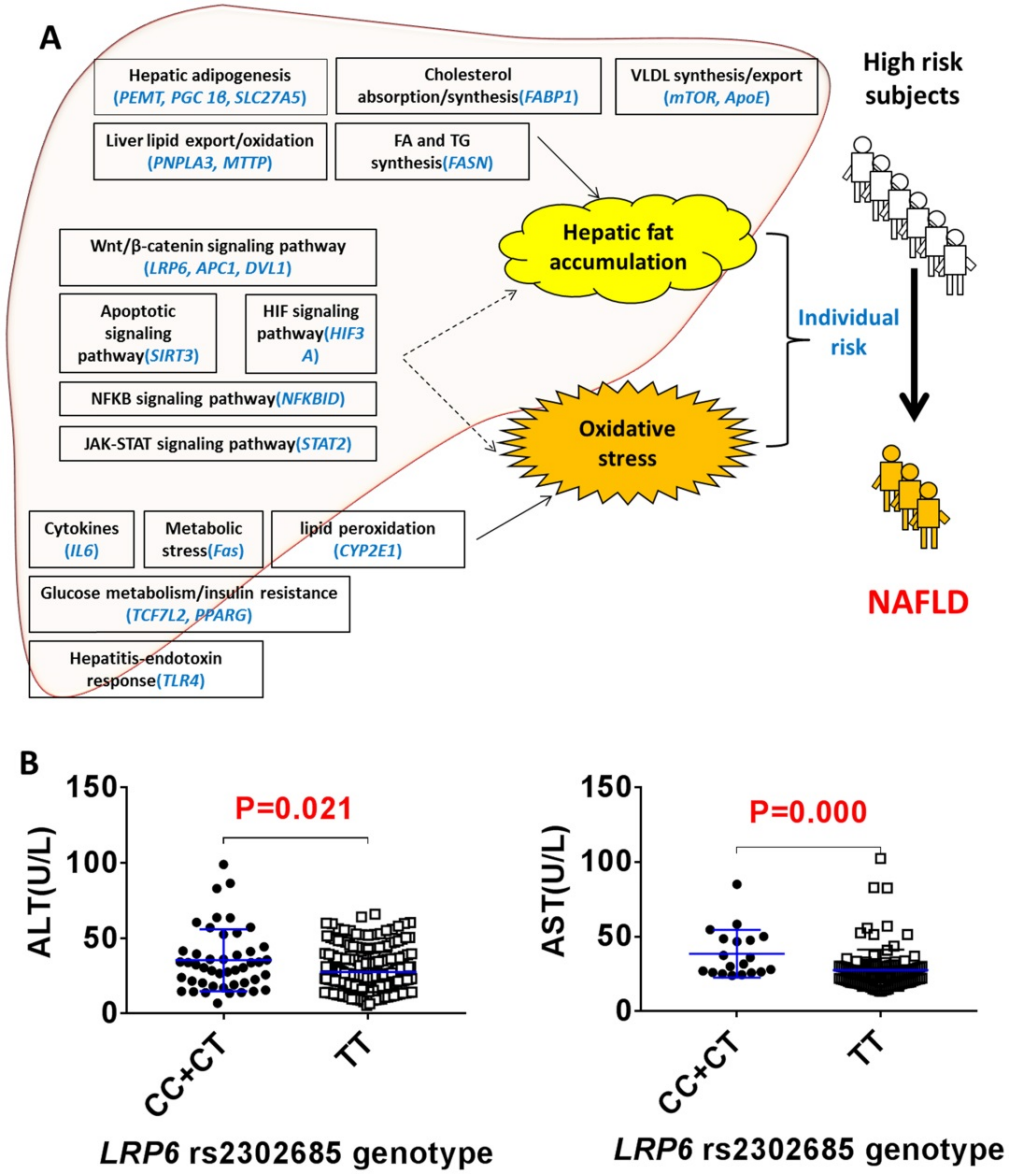
***LRP6 rs2302685* was associated with a reduced liver injury in NAFLD patients. (A)** Distribution of 22 SNPs likely contributing to the individual risk of NAFLD based on the 'two-hit hypothesis'. **(B)** Comparison of the serum levels of ALT and AST between the *LRP6 rs2302685* T allele carriers (n=47) and C allele carriers (n=239) in NAFLD subjects.

The clinical protocol for screening NAFLD and non-NAFLD subjects is shown in additional file **Figure S1**. We recruited 292 NALFD and 387 non-NAFLD eligible subjects. The demographics and clinical characteristics between the NAFLD and non-NAFLD subjects are shown in **Table 1**. We genotyped 22 SNPs of 22 genes in various physiological processes related to the 'two-hit hypothesis': the hepatic lipid import/synthesis (*PEMT*, *PGC 1β*, *SLC27A5*), hepatic lipid export/oxidation (*PNPLA3*, *MTTP*), cholesterol absorption/synthesis (*FABP1*), fatty acid/triglyceride synthesis (*FASN*), VLDL synthesis/export (*ApoE*, *mTOR*), cytokines (*IL6*), metabolic stress (*Fas*), lipid peroxidation (*CYP2E1*), steatohepatitis-endotoxin response (*TLR4*), glucose metabolism/insulin resistance (*TCF7L2*, *PPARG*), Wnt/β-catenin signaling pathway (*LRP6*, *APC1*, *DVL1*), apoptosis signaling pathway (*SIRT3*), HIF signaling pathway (*HIF3A*), NFKB signaling pathway (*NFKBID*) and JAK-STAT signaling pathway (*STAT2*) (**Figure 1A**). As shown in additional file **Table S1**, the odd ratios for NAFLD between the different SNP genotypes of *rs2241883* (*FABP1*) and *rs2302685* (*LRP6*) were found to be significantly different in the population. The T allele of* LRP6 rs2302685* was significantly associated with a higher risk (OR=2.853; 95% CI, 1.378-5.910; P=0.005) of NAFLD in comparison to the C allele in subjects, while *FABP1 rs2241883* (T allele) was significantly associated with a lower risk (OR=0.606; 95% CI, 0.380-0.968; P=0.036) of NAFLD. However, the plasma levels of aspartate aminotransferase (AST) and alanine transaminase (ALT) were 28.6% and 21.9% lower in the NAFLD subjects carrying *LRP6 rs2302685* T allele than those carrying the C allele, respectively (**Figure 1B**, **Table 2**), suggesting differential roles of LRP6 in NAFLD risk and its liver injury. No significant differences in AST and ALT levels were found between *FABP1 rs2241883* T and C alleles.

### *Lrp6*^(+/-)^ genotype ameliorated liver injury in NAFLD mice

**Figure 2:**
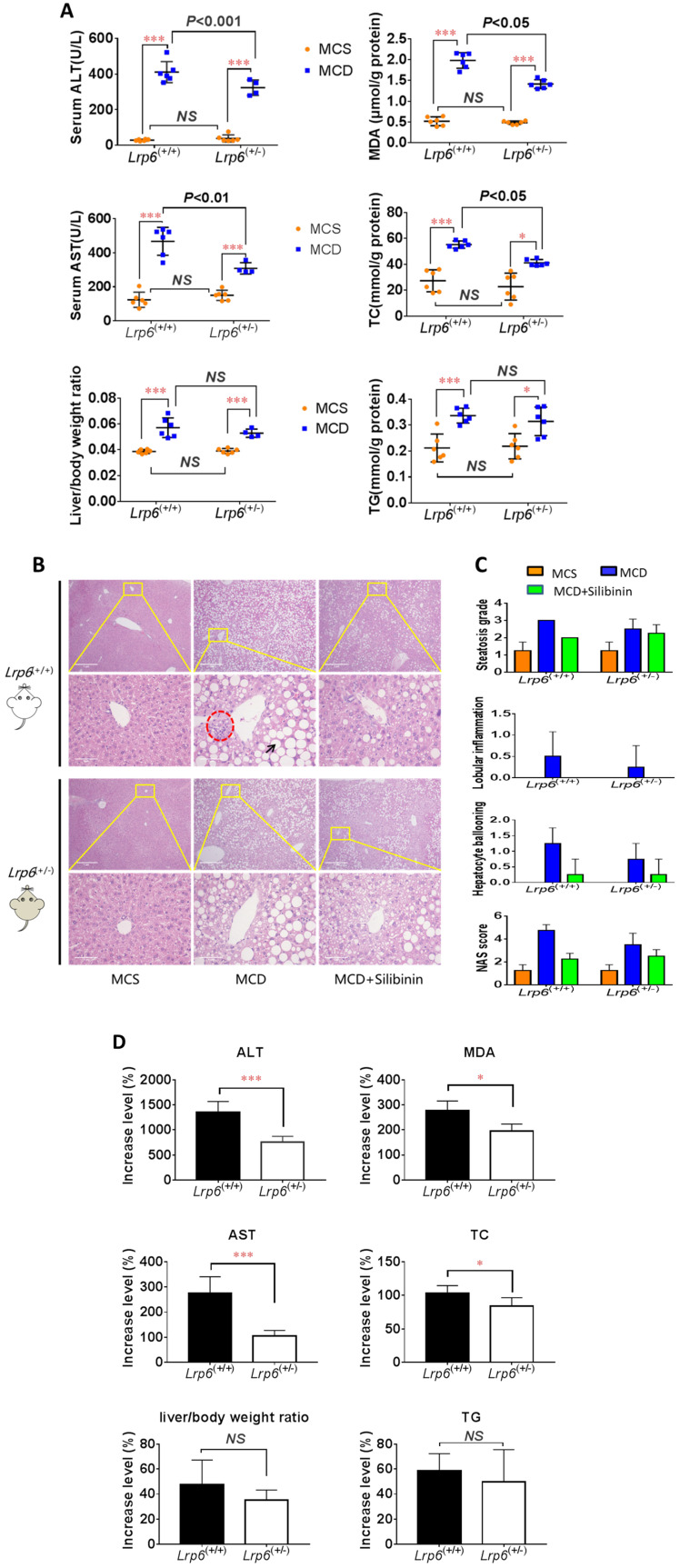
***Lrp6* haploinsufficiency (*Lrp6*^(+/-)^) ameliorated liver injury and reduced the efficacy of silibinin in NAFLD mice.** (A) Comparison of liver function, as determined from the levels of enzymatic markers, and liver/body weight ratio between the *Lrp6*^(+/+)^ and the *Lrp6*^(+/-)^ mice received MCD diet. The MCD diet was used to induce liver injury and NAFLD in mice. (B) Effect of *Lrp6* genotypes on liver histological injury in mice received different diets and treatments. The histology of liver tissues was examined after H&E staining. The macrovesicular steatosis was indicated with arrows, while necroinflammatory foci were indicated with circular broken lines (Scale bar, 1μM for the above row, and 5μM for the below row in each group). (C) Effects of diets MCS and MCD on liver injury in mice. The hepatic steatosis, hepatocyte ballooning, lobular inflammation and NAS score were examined under microscopy and compared between the *Lrp6*^(+/+)^ and the *Lrp6*^(+/-)^ mice with and without silibinin treatment. (D) Comparison of the change rate for injury indicators between the *Lrp6*^(+/+)^ and the *Lrp6*^(+/-)^ mice groups. * *P* < 0.05; ** *P* < 0.01; *** *P* < 0.001; NS, No significance.

To ascertain a role of LRP6 in the liver injury of NAFLD, we conducted animal studies using genetic mouse models as outlined in additional file**Figure S2**. The MCD diet was used to induce NAFLD in mice. The mice body weight and low density lipoprotein cholesterin (LDL-c) were significantly decreased in both *Lrp6*^(+/-)^ and *Lrp6*^(+/+)^ mice that received MCD diet as compared to those received the control MCS diet (Additional file**Figure S3, S4 and S5**). However, the *Lrp6* genotype had no significant effect on body weight (Additional file**Figure S3**). The levels of biomarkers for liver injury, including ALT, AST and liver/body ratio, were all significantly increased in the MCD diet groups when compared with the corresponding MCS diet control groups. In addition, the MCD diet significantly increased the hepatic levels of MDA, which reflected oxidative stress, TG and TC, which were related to fat accumulation in the liver tissues (**Figure 2A**, **Table 3**). H&E staining also showed that liver injury was more severe in the MCD diet groups than in the MCS control groups (**Figure 2B and 2C**). These results confirmed that the MCD diet could reliably induce NAFLD in mice.

The severity of NAFLD, as reflected by those liver injury biomarkers and metabolic measurements, were further compared between the two *Lrp6* genotypes of mice. The increase of ALT, AST, TC, and MDA by MCD diet in *Lrp6*^(+/-)^ mice were 598.7% (P=0.0009), 169.9% (P=0.0012), 19.23%(P=0.0429) and 82.6% (P=0.0185) significantly lower than those in the *Lrp6*^(+/+)^ mice, respectively. While no significant differences in MCD-induced increase of TG and liver/body ratio were found, the *Lrp6*^(+/-)^ mice tended to have a decreased level of hepatic TG and a smaller liver/body ratio as compared to the *Lrp6*^(+/+)^ mice (**Figure 2D**). The liver injury by H&E staining in the *Lrp6*^(+/-)^ mice was relatively mild when compared with that in the *Lrp6*^(+/+)^ mice (**Figure 2B and 2C**). In summary, the haploinsufficiency of *Lrp6* (*Lrp6*^(+/-)^) alleviated MCD diet-induced liver injury and NAFLD in mice.

### *Lrp6*^(+/-)^ genotype reduced the therapeutic effects of silibinin in NAFLD mice

**Figure 3:**
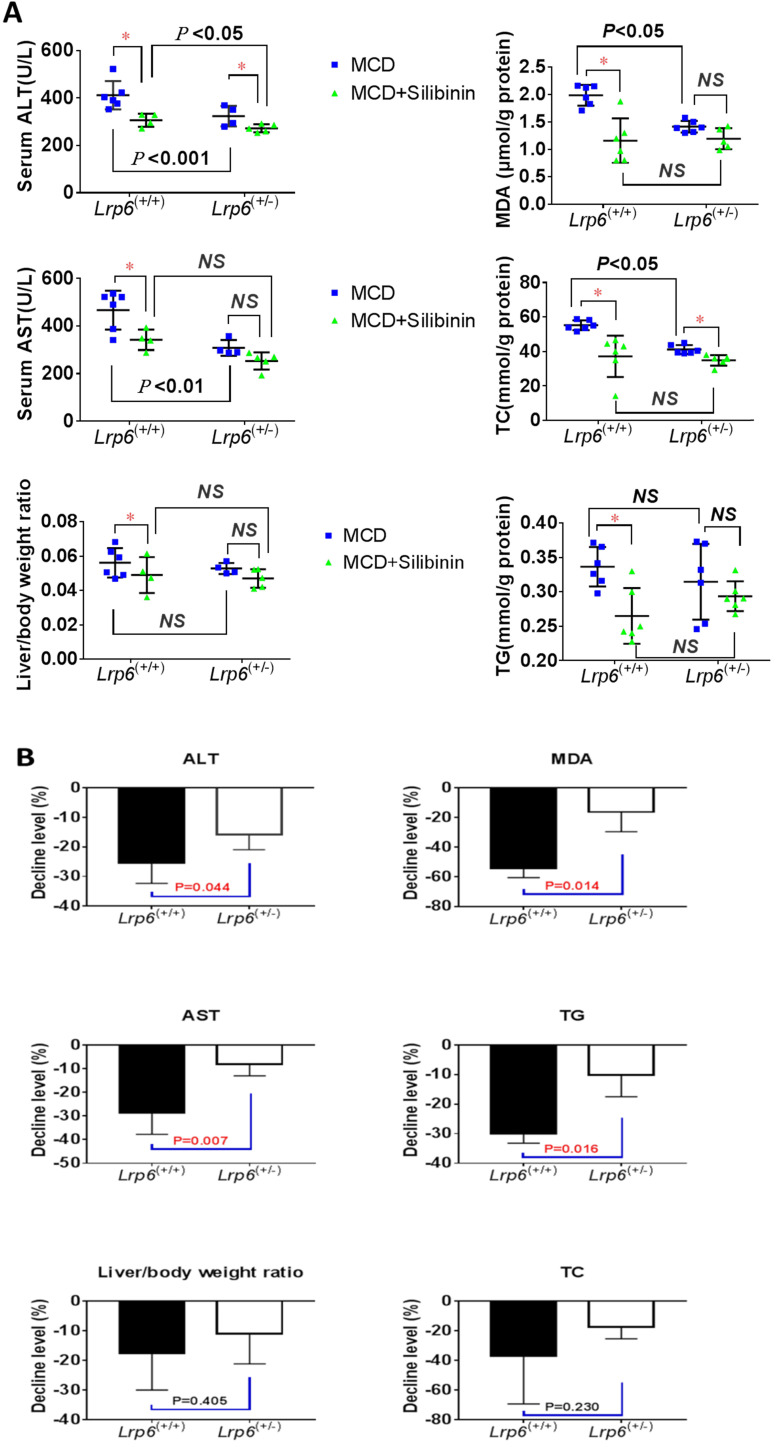
***Lrp6* haploinsufficiency (*Lrp6*^(+/-)^) affected the therapeutic effect of silibinin in NAFLD mice. (A)** Comparison of the liver function, as determined from the levels of enzymatic markers, and liver/body weight ratio between the *Lrp6*^(+/+)^ and the *Lrp6*^(+/-)^ mice received MCD diet with and without the treatment of silibinin. **(B)** Change of the liver function and liver/body weight ratio by silibinin treatment between the *Lrp6*^(+/+)^ and *Lrp6*^(+/-)^ mice. * *P* < 0.05; NS, No significance.

We investigated the therapeutic effects of silibinin against NAFLD and the impact by *Lrp6* genotypes in mice. The administration of Silibinin (100 mg/kg/d) caused improvement on the levels of most of the liver injury biomarkers, including ALT, AST, MDA, TG and TC, that had been induced by the MCD diet in mice (**Figure 3A**, **Table 3**), as well as the degree of liver injury shown by the H&E staining (**Figure 2B and 2C**). The results suggested that while silibinin treatment might not completely stop the progress of NAFLD, it could reduce the liver injury associated with NAFLD.

The therapeutic effects of silibinin on NAFLD was also compared between *Lrp6*^(+/-)^ and *Lrp6*^(+/+)^ mice. The degrees of ALT, AST, MDA and TG reduced by silibinin treatment in the *Lrp6*^(+/-)^ mice were significantly lower (from 9.7-38.2%) than those in the *Lrp6*^(+/+)^ mice (**Figure 3B**). Consistently, the decrease of liver fat accumulation and degree of liver injury in the H&E staining in *Lrp6*^(+/-)^ mice were relatively less significant as compared to those in the *Lrp6*^(+/+)^ mice (**Figure 2B and 2C**). Taken together, while the haploinsufficiency of *Lrp6* could reduce the liver injury associated with NAFLD, it led to a reduced therapeutic response to silibinin treatment on the disease, suggesting Lrp6 as a target for silibinin treatment.

### Lrp6 was a target for silibinin to inhibit the Wnt/β-catenin-Cyp2e1 signaling pathway and the generation of ROS

**Figure 4:**
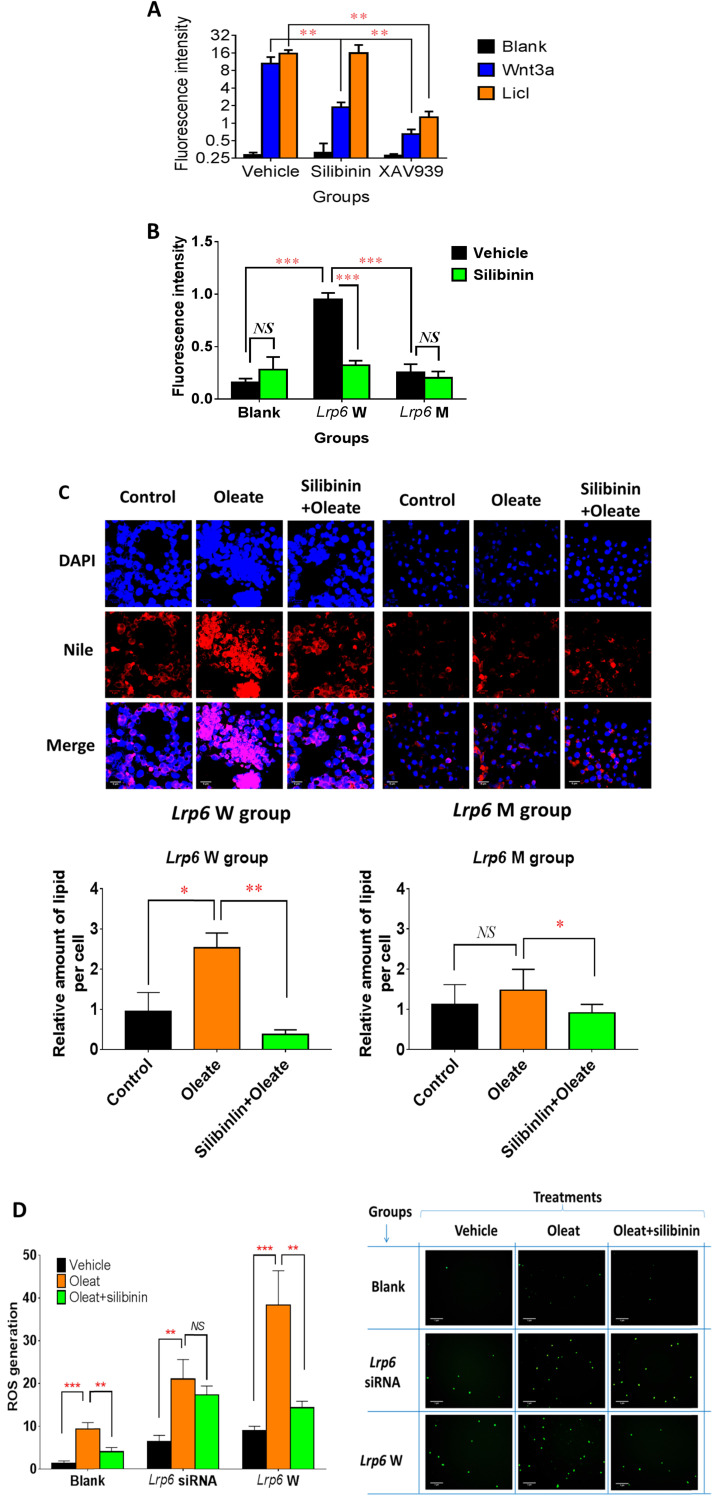
** Lrp6 was a target for silibinin to inhibit the Wnt/β-catenin-Cyp2e1 signaling pathway and the generation of ROS. (A)** Comparison of the Wnt/β-catenin signaling activity between the silibinin-treated and untreated HL7702 cells. The Wnt/β-catenin signaling activity was determined from the fluorescence intensity induced by LiCl treatment or Wnt3a-conditioned medium in reporter gene assays. XAV939, a characterized Wnt/β-catenin pathway inhibitor, served as a positive control. **(B)** Comparison of the Wnt/β-catenin signaling activity between the silibinin-treated and untreated TOPFlash HEK293 cells overexpressing *Lrp6* W (wide-type) or *Lrp6* M (mutant). The Wnt/β-catenin signaling activity was determined by reporter gene assays for the TOPFlash cells, as described in Methods. **(C)** Comparison of the oleat uptake between the silibinin-treated and untreated HL7702 cells overexpressing *Lrp6* W (wide-type) or *Lrp6* M (mutant) (Scale bar, 5μM). **(D)** Comparison of ROS generation induced by oleat treatment between the silibinin-treated and untreated HL7702 cells transfected with *Lrp6* siRNA or *Lrp6* W vector (Scale bar, 1μM). * P < 0.05; ** *P* < 0.01; *** *P* < 0.001; NS, No significance.

Because of the critical role of Lrp6 in the Wnt/β-catenin pathway, we studied the effect of silibinin treatment on the signaling activity of this pathway in cell models. Compared with the vehicle control groups, silibinin significantly inhibited the Wnt/β-catenin signaling activity, as determined from the fluorescence intensity induced by Wnt3a- (a potent agonist for Lrp6) conditioned DMEM medium in reporter gene assays, by 82.18%. Interestingly, silibinin did not affect the Wnt/β-catenin signaling activity induced by 25 mM LiCL (specifically inhibit the degradation of β-catenin by inhibiting the activity of GSK-3β). As a positive control, XAV 939 stimulates β-catenin degradation, resulting in the inhibition of the Wnt/β-catenin pathway. XAV 939 inhibited Wnt3a- and LiCl-induced Wnt/β-catenin signaling activity by 93.86% and 91.94%, respectively (**Figure 4A**). Furthermore, the overexpression of *Lrp6* W (wild-type) significantly increased the Wnt/β-catenin signaling activity by 499.84% when compared with the empty vector control, which could be significantly reduced by silibinin treatment. In contrast, the overexpression of *Lrp6* M (mutation) had no effect on the Wnt/β-catenin signaling activity (**Figure 4B**). In further functional studies, oleat treatment could significantly enhance lipid accumulation in HL7702 cells overexpressing *Lrp 6* W, while no apparent oleat-induced lipid accumulation was detected in those overexpressing *Lrp6* M (**Figure 4C**). In particular, in the presence of *Lrp6* W overexpression, silibinin treatment could fully block the lipid accumulation induced by oleat treatment in HL7702 cells. However, in the presence of *Lrp6* M overexpression, silibinin treatment did not have any effect (**Figure 4C**). In addition, oleat treatment significantly induced ROS generation, especially with the overexpression of the *Lrp6* W. Silibinin treatment could significantly reduce ROS generation induced by oleat treatment. However, the effect of silibinin treatment on ROS generation was insignificant in the cells pre-treated *Lrp6* siRNA (**Figure 4D**). Together, our data indicated that Lrp6 was the action site for silibinin to inhibit activity of Wnt/β-catenin signaling pathway and the consequent ROS generation.

### *Lrp6* expression and silibinin treatment affected the expression and activity of Cyp2e1 through the Wnt/β-catenin signaling pathway

**Figure 5:**
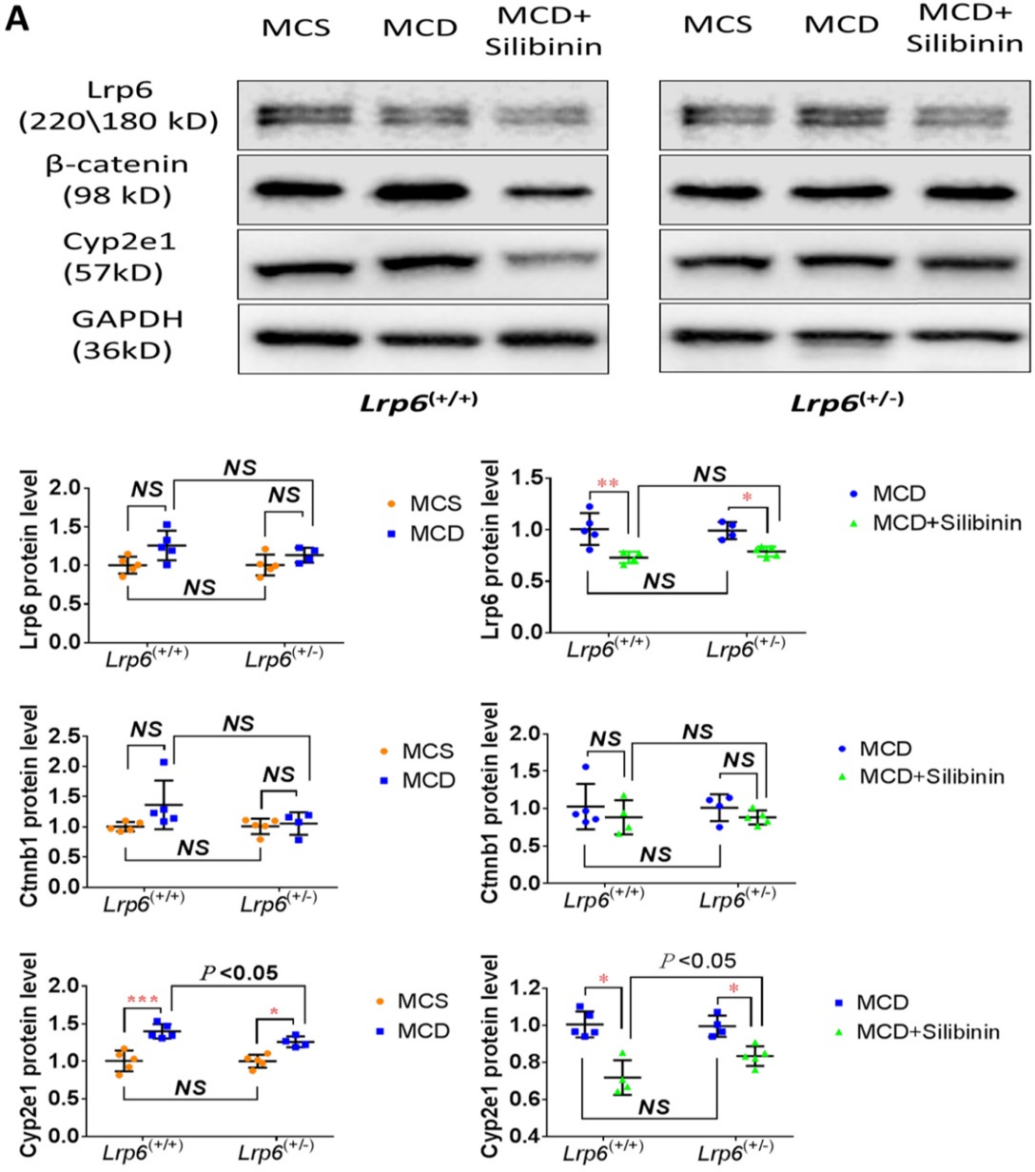

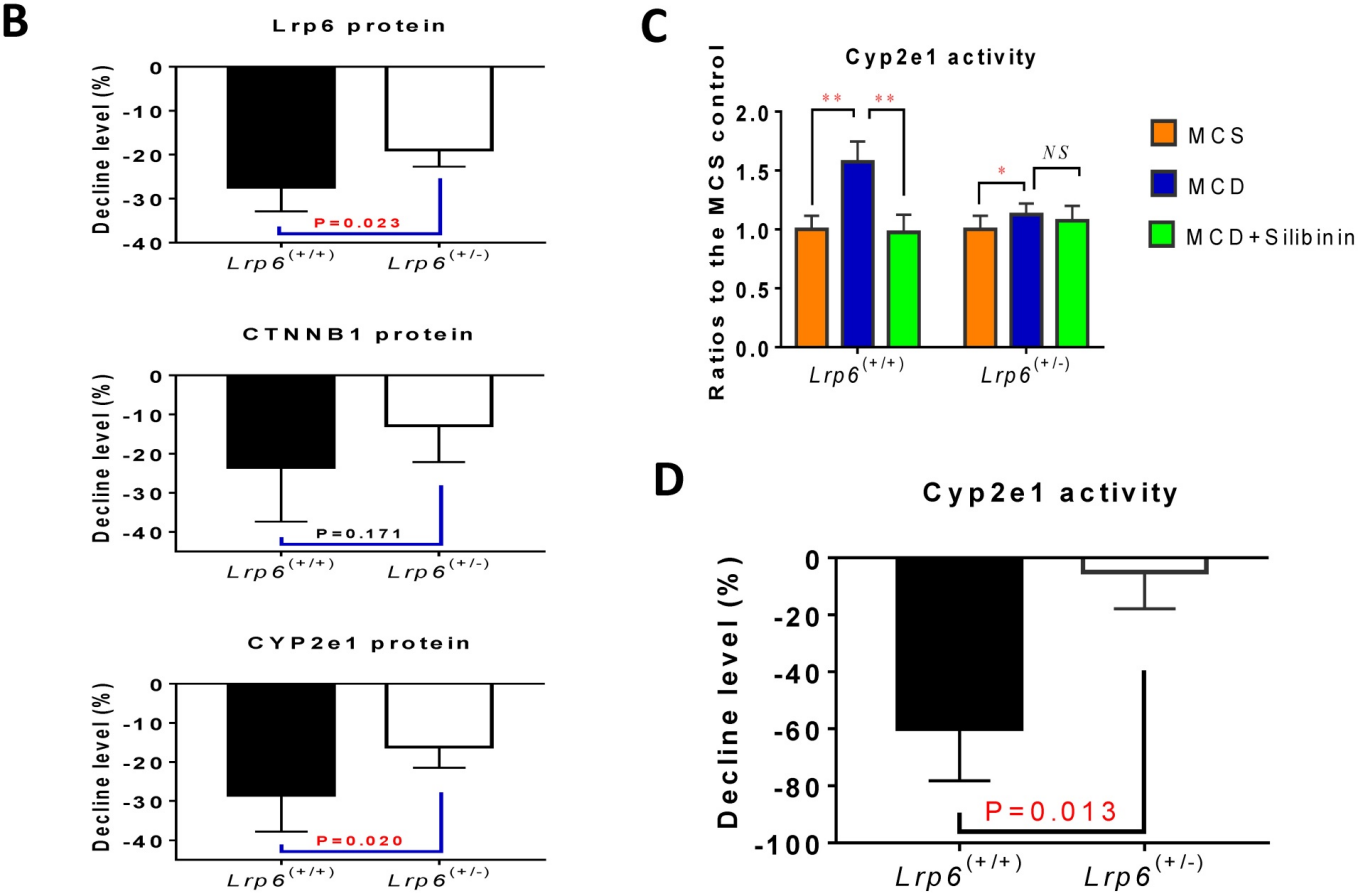

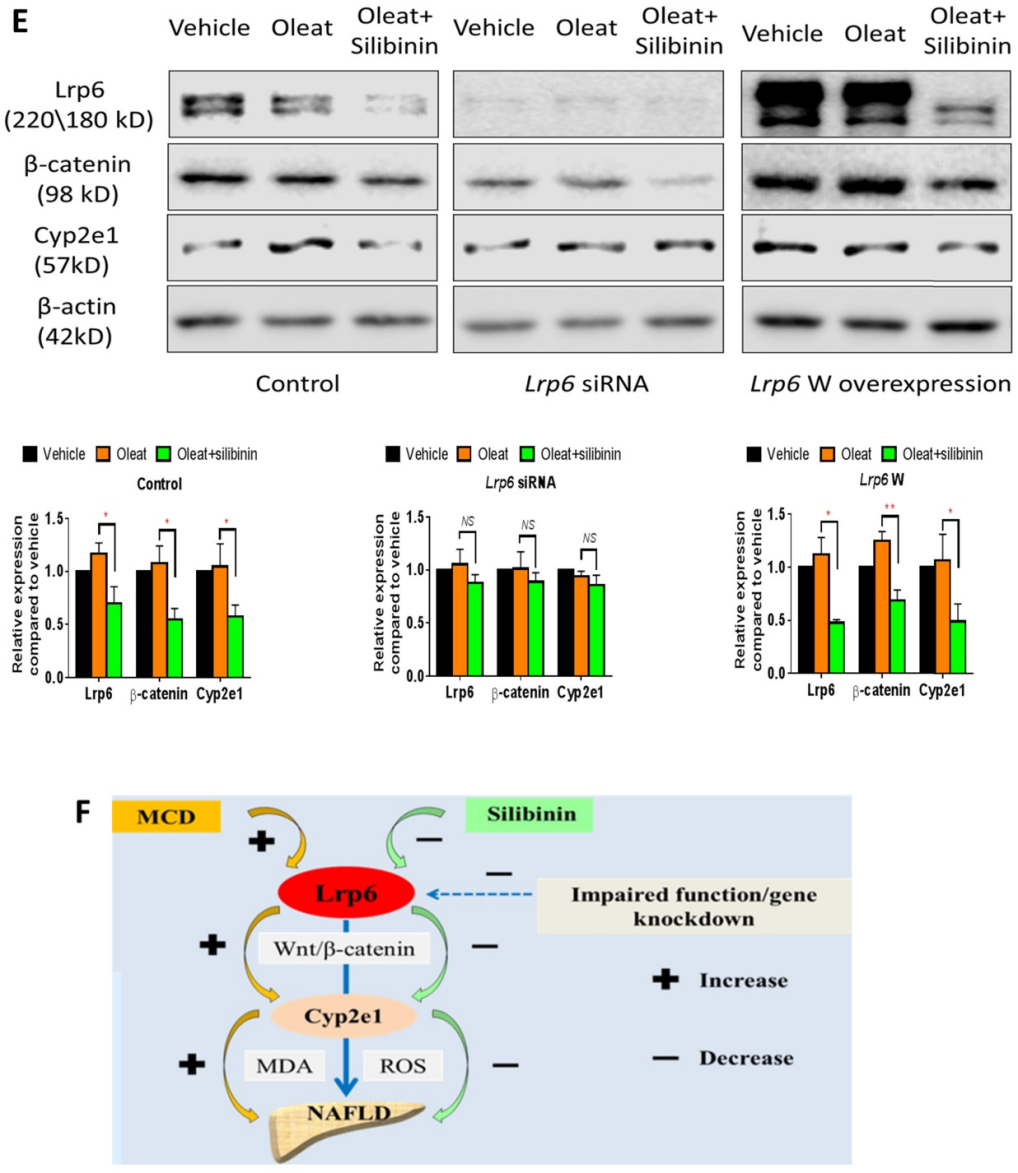
** Effects of *Lrp6* expression and silibinin treatment on the expression and activity of Cyp2e1. (A)** Protein expression of Lrp6, β-catenin and Cyp2e1 among different treatment groups in mice. **(B)** Expression change of Lrp6, β-catenin and Cyp2e1 proteins by silibinin treatment between the *Lrp6*^(+/+)^ and the *Lrp6*^(+/-)^ mice. **(C)** Effects of Lrp6 genotypes and silibinin treatment on the activity of Cyp2e1 in mice received MCD diet. **(D)** The activity change of Cyp2e1 by silibinin treatment between the *Lrp6*^(+/+)^ and *Lrp6*^(+/-)^ mice. **(E)** Effects of Lrp6 expression on the expression changes of Lrp6, β-catenin and Cyp2e1 in HL7702 cells with and without oleat treatment. The cells were transfected with a control vector, the vectors containing Lrp6 siRNA or Lrp6 W. **(F)** The scheme showing that *Lrp6* functional alteration confers different susceptibility to NAFLD and affects the therapeutic effect of silibinin by modulating Wnt/β-catenin-CYP2E1 signaling. * *P* < 0.05; ** *P* < 0.01; *** *P* < 0.001; NS, No significance.

The activity of Cyp2e1 has been frequently reported to play an important role in oxidative stress and associated with the development of NAFLD in previous studies 32. To explain why Lrp6 haploinsufficiency ameliorated liver injury and reduced the efficacy of silibinin in MCD diet-induced NAFLD, we compared the hepatic expression of Cyp2e1 and the key proteins including Lrp6 and β-catenin, in Wnt/β-catenin signaling between *Lrp6*^(+/-)^ and *Lrp6*^(+/+)^ mice received either MCD or MCS diet. Firstly, the expression of Lrp6, β-catenin and Cyp2e1 in the liver was found to be significantly upregulated in both *Lrp6* genotypes of mice with the MCD diet-induced NAFLD relative to those received the MCS diet, respectively (**Figure 5A**), confirming a role by these proteins in NAFLD.

Moreover, silibinin treatment significantly reduced the hepatic expression of Lrp6, β-catenin and Cyp2e1 induced by the MCD diet in both *Lrp6* genotypes of mice (**Figure 5A**). Of note, the expression of Cyp2e1 reduced by silibinin treatment in the liver of *Lrp6*^(+/-)^ mice was significantly less than what was found in the liver of *Lrp6*^(+/+)^ mice (*P* = 0.023) (**Figure 5B**). In addition, the MCD diet significantly increased the activities of Cyp2e1 in both *Lrp6*^(+/-)^ and *Lrp6*^(+/+)^ mice (**Figure 5C**). Silibinin treatment apparently reduced the activity of liver microsomal Cyp2e1 in the *Lrp6*^(+/+)^ mice, but not in the *Lrp6*^(+/-)^ mice (60.0% *vs.* 5.4%, *P* = 0.013) (**Figure 5D**). To validate the above mouse findings, we also conducted studies in HL7702 cells. In oleat-treated HL7702 cells, silibinin treatment could significantly reduce the expression of Lrp6, β-catenin and Cyp2e1 that was increased by Lrp6 overexpression (**Figure 5E**). However, the effect of silibinin treatment on the expression of these proteins was abolished by Lrp6 siRNA.

Overall, our results indicated that a loss of *Lrp6* function, such as *rs2302685* in human subjects, *Lrp6* haploinsufficiency in mice, and *Lrp6* knockdown by siRNA in cells, was associated with hypofunction of Wnt/β-catenin-Cyp2e1 signaling, which resulted in a lower degree of liver injury associated with NAFLD as well as a reduced response to silibinin treatment (**Figure 5F**).

## Discussion

In the present study, *LRP6 rs2302685* was confirmed to be associated with a risk of NAFLD in human subjects. We then employed *Lrp6*^(+/-)^ mice to model a potential partial function loss of *LRP6* caused by *rs2302685*. In the follow-up animal studies, the *Lrp6*^(+/-)^ mice were found to be less susceptible to liver injury induced by MCD diet when compared to wild-type mice. However, the therapeutic efficacy of silibinin against MCD-induced NAFLD was reduced in the *Lrp6*^(+/-)^ mice. Further cellular and molecular analyses indicated that the Wnt/β-catenin-Cyp2e1 signaling pathway mediated the production of ROS that was highly related to the liver injury associated with NAFLD progress. The altered activity of Wnt/β-catenin-Cyp2e1 pathway associated with human *rs2302685* and mouse *Lrp6* haploinsufficiency might account for the increased susceptibility to NAFLD and the reduced response to silibinin treatment. The present study has therefore demonstrated a role by *LRP6* in the development of NAFLD and its drug treatment, offering Wnt/β-catenin-Cyp2e1 pathway as the underlying mechanism for the genetic risk of NAFLD associated with* rs2302685* and as a target for the treatment of NAFLD.

Lrp6 is an important member of the low-density lipoprotein receptor family and a key regulatory protein at the upstream of the Wnt/β-catenin signaling pathway 15. The Wnt/β-catenin signaling pathway has critical physiological and pathological functions, through which a large number of target genes for cell proliferation, differentiation and tumorigenesis are transcriptionally regulated 33. *LRP6* rs2302685, a common functional germline variant (MAF≈15%), has been previously linked to many other diseases. For example, it is a known risk factor for carotid atherosclerosis in patients with hypertension and has been found to significantly increase the incidence of myocardial infarction in the Chinese Han population 18, 34. Some studies reported that it was a risk factor for the late-onset Alzheimer's disease 35. The polymorphism was also associated with the risk of hyperlipidaemia in Iranian children and adolescents 17. In addition, we have previously reported that *LRP6 rs2302685* contributes to individual susceptibility to Alcoholic Liver Disease (ALD) 19. However, no previous studies have reported any association between *LRP6 rs2302685* and NAFLD. Given the high frequency of *rs2302685* and high prevalence of NAFLD in human populations, the significant association between* rs2302685* and NAFLD identified in our studies has important clinical implication.

Many other genetic variants have already been reported as contribution to the individual susceptibility to NAFLD. The *rs2241883* (T>C, T94A) in the gene for the liver fatty acid binding protein (*L-FABP*) is such a variant. L-FABP is thought to promote an early adaptive response to stress by affecting fatty acid uptake and intracellular esterification in lipid pools in hepatocytes 36. In the present study, the T allele of *L-FABP* rs2241883 was also found to be associated with a reduced risk of NAFLD, The mechanism underlying this association remains to be further illustrated. It is worth mentioning that *PNPLA3* rs738409 (C>G), a SNP discovered from a Genome-wide Association Study for NAFLD, has been widely reported as a very strong genetic determinant for the levels of liver fat and ALT 37, 38, however, we were unable to establish any significant association between *rs738409* and NAFLD or the measurements on liver function in human subjects in the present study. We also studied other representative SNPs in the genes related to the 'two-hit hypothesis', such as phosphatidylethanolamine-N-methyltransferase (*PEMT*) 10, peroxisome proliferator-activated receptor gamma coactivator 1β (*PGC 1β*) 39, *LPIN1* 40, solute carrier family 27 member 5 (*SLC27A5*), microsomal triglyceride transfer protein (*MTTP*) 41, fatty acid synthase (*FASN*) 42, apolipoprotein E (*ApoE*) 43, cytochrome P450 2E1 (*CYP2E1*) 32, cytochrome P450 4A11 (*CYP4A11*) 25, glucokinase regulator (*GCKR*) 44, transcription factor 7 like 2 (*TCF7L2*), peroxisome proliferator-activated receptor gamma (*PPARG*) 45, 46, toll-like receptor 4 (*TLR4*) 47, transforming growth factor beta 1(*TGFβ1*) 48, 49 and interleukin 6 (*IL6*) 50.

These SNPs have been reported as being associated with the individual susceptibility to NAFLD in previous studies. However, none of them was found to be associated with NAFLD in the present study. Whereas the difference outcomes could have been explained by various factors such as study regions and ethnicities 4, it underlies the importance of replication for those association studies by different populations.

Interestingly, the therapeutic effects of silibinin on NAFLD mice were reduced by *Lrp6* haploinsufficency in the present study. Although a clinical study is warranted, our cellular and animal findings suggested that the human subjects carrying different *Lrp6* genotypes might have different response to silibinin treatment. In fact, Lrp6 has been previously identified as a novel therapeutic target for several liver diseases, including NAFLD and hyperlipidaemia 15, 51. Overall, the Lrp6-Wnt/β-catenin signaling pathway has already been widely explored in drug development 52. Considering that *LRP6 rs2302685* occurs in about 15% of the population 18, we need to pay attention to the potential variation in drug response that is associated with the genetic polymorphism. For example, in the development of drugs targeting Lrp6-Wnt/β-catenin pathway, the genotype-based patient stratification may be necessary to design clinical trials. In clinical practice, the genetic polymorphisms in the Lrp6-Wnt/β-catenin pathway may have an important impact on precision medicine while prescribing those drugs with an action site in this pathway, such as silibinin.

Our data suggested that CYP2E1 might be a critical effector in the downstream of LRP6-Wnt/β-catenin pathway accounting for the reduced liver injury as well as silibinin response associated with Lrp6 function loss. CYP2E1, a key player in cellular oxidative stress and metabolism, is one of the target proteins subject to the regulation by Wnt/β-catenin signaling pathway 21, 29. CYP2E1 belongs to the cytochrome P450 super family that is a group of heme-containing proteins responsible for the metabolism of xenobiotics, such as drugs, toxins, carcinogens and endogenous substrates. CYP2E1 also metabolises polyunsaturated fatty acids such as linoleic acid and arachidonic acid. The expression of CYP2E1 could be induced by certain exogenous substrates such as ethanol as well as certain endogenous substrates such as polyunsaturated fatty acids. CYP2E1 plays a role in the liver injury in NAFLD as it is involved in the biochemical reactions by generating ROS and lipid peroxidation products 19. The reduction of endogenous antioxidants in ALD and NAFLD may enhance CYP2E1-induced lipid peroxidation, oxidant stress and cellular toxicity. Increased CYP2E1 protein expression and activity were often found in obesity, fatty liver and NASH in both humans and rodents 29, 32. In the present study, when compared with the wide-type mice, the* Lrp6*^(+/-)^ mice had less liver injury induced by the MCD diet, with the lower expression and activity of Cyp2e1 as well as the reduced amount of tissue ROS. Our data also supports Lrp6 as a target of silibinin. Lrp6 knockdown could reduce the inhibitory effect of silibinin on Wnt/β-catenin activity, resulting in a lower absorption of oleate and a lower level of ROS generation in cells. Interestingly, the expression and activity of Cyp2e1 were both significantly reduced by the treatment of silibinin. Consistently, silibinin has been previously reported as being an inhibitor for Wnt/β-catenin signaling that could reduce the expression of Lrp6 53. Whereas the LRP6-Wnt/β-catenin-Cyp2E1 signaling pathway has been demonstrated as a contributing mechanism for the liver injury and silibinin response in NAFLD in the present study, future studies are needed to determine how important CYP2E1 is.

## Conclusions

In conclusion, we have demonstrated that the function loss of *Lrp6*, either likely *via* a germline variant in human subjects or through genetic manipulation in rodents or cell models, can result in a less severity of NAFLD, This may be explained by the consequent downregulation of the Wnt/β-catenin-Cyp2e1 signaling activity. The present work has provided an underlying mechanism for human individual susceptibility to NAFLD associated with *Lrp6 rs2302685* as well as rational usage of silibinin in clinical settings.

## Supplementary Material

Supplementary figures and table.

## Figures and Tables

**Table 1:** The demographics and clinical characteristics for NAFLD and Non-NAFLD subjects

Characteristic	NAFLD (n=292)	Non-NAFLD (n=387)	P Value
Age, year	48.89±11.57	41.97±12.17	**0.000**
Gender, Male/Female	177/114	134/251	**0.000**
BMI, kg/m^2^	26.25±2.55	22.13±2.56	**0.000**
SBP, mmHg	123.95±19.82	119.32±0.96	0.282
DBP, mmHg	77.85±0.86	75.80±0.65	0.089
WC, cm	79.32±0.42	77.56±0.32	**0.003**
HP, cm	91.60±0.36	92.05±0.27	0.366
TP, g/L	73.83±0.29	73.56±0.22	0.497
Albumin, g/L	46.26±0.24	45.80±0.18	0.159
Globulin, g/L	28.10±0.36	27.79±0.27	0.538
A/G	1.70±0.02	1.69±0.02	0.575
TBIL, μmol/L	12.59±0.60	13.14±0.45	0.502
DBIL, μmol/L	5.39±0.22	5.23±0.17	0.606
TBA, μmol/L	3.69±0.28	3.05±0.25	0.137
ALT, U/L	30.77±1.70	20.60±1.29	**0.000**
AST, U/L	26.42±1.46	24.30±1.31	0.339
FBG, mmol/L	5.47±0.11	5.17±0.09	0.055
LDL-C, mmol/L	3.041±0.06	2.88±0.05	0.068
TG, mmol/L	2.25±0.10	1.51±0.08	**0.000**
TC, mmol/L	5.18±0.07	4.95±0.06	**0.025**
HDL-C, mmol/L	1.40±0.03	1.52±0.02	**0.001**

**Note:** Values are expressed as mean±SD and compared by analysis of covariance corrected for Age, Gender and BMI, except for Age and BMI by t test(values are expressed as mean±SD) and gender by Chi-square test(values are expressed as n). A *P-value* < 0.05 was considered as statistically significant (in bold). **Abbreviations:** BMI, Body Mass Index; SBP, systemic blood pressure; DBP, diastolic blood pressure; WC, Waist circumference; HP, Hip circumference; TP, Total Protein; A/G, the ratio of albumin to globulin; TBIL, total bilirubin; DBIL, direct bilirubin; TBA, total bile acid; ALT, glutamic-pyruvic transaminase; AST, glutamic oxalacetic transaminase; FBG, fasting blood-glucose; LDL-C, low density lipoprotein cholesterin; TG, triglyceride; TC, total cholesterol; HDL-C, high density lipoprotein cholesterol; NAFLD, nonalcoholic fatty liver disease.

**Table 2:** The comparison of demographics and clinical characteristics between *LRP6 rs2302685* CC /CT and TT genotypes in NAFLD subjects

Characteristic	CC/CT genotype (n=47)	TT genotype (n=239)	P value
Age, year	45.8±11.26	49.62±11.54	0.019
Gender, Male/Female	32/15	141/97	0.257
BMI, kg/m^2^	26.94±2.22	26.11±2.6	0.030
SBP, mmHg	124.56±22.72	123.62±19.19	0.769
DBP, mmHg	84.37±14.33	84.07±15.97	0.702
WC, cm	85.26±7.53	85.45±8.51	0.960
HP, cm	96.91±5.5	95.01±6.91	0.018
TP, g/L	72.18±5.37	72.48±4.85	0.819
Albumin, g/L	45.17±4.85	44.94±4.53	0.217
Globulin, g/L	27.01±3.06	27.81±5.07	0.241
A/G	1.69±0.28	1.67±0.35	0.307
TBIL, μmol/L	13.91±8.7	12.5±5.78	0.580
DBIL, μmol/L	5.95±4.08	5.34±3.37	0.496
TBA, μmol/L	4.56±2.74	4.5±5.33	0.220
**ALT, U/L**	**35.43±20.53**	**27.67±13.34**	**0.021**
**AST, U/L**	**38.59±16.01**	**27.57±13.74**	**0.000**
FBG, mmol/L	5.21±0.61	5.63±1.77	0.557
LDL-C, mmol/L	2.97±0.89	3.16±0.83	0.140
TG, mmol/L	2.59±2.43	2.5±1.72	0.914
TC, mmol/L	5.02±1.1	5.18±1.06	0.320
HDL-C , mmol/L	1.22±0.34	1.28±0.32	0.591

**Note:** Values are expressed as mean±SD and compared by Student's t-test if the data is normal distribution, otherwise Mann-Whitney U test is used, except for gender that p value stands for statistical significance using Chi-square test. A *P*-value < 0.05 was considered as statistically significant (in bold). **Abbreviations:** NAFLD, nonalcoholic fatty liver disease; BMI, Body Mass Index; SBP, systemic blood pressure; DBP, diastolic blood pressure; WC, Waist circumference; HP, Hip circumference; TP, Total Protein; A/G, the ratio of albumin to globulin; TBIL, total bilirubin; DBIL, direct bilirubin; TBA, total bile acid; ALT, glutamic-pyruvic transaminase; AST, glutamic oxalacetic transaminase; FBG, fasting blood-glucose; LDL-C, low density lipoprotein cholesterin; TG, triglyceride; TC, total cholesterol; HDL-C, high density lipoprotein cholesterol.

**Table 3:** Morphological and biochemical changes in mice with different *Lrp6* genotypes under different treatments

Parameters	Lrp6^(+/+)^ genotype			Lrp6^(+/-)^ genotype		
	MCS	MCD	MCD+Silibinin	MCS	MCD	MCD+Silibinin
Liver body ratio (%)	3.87±0.16	5.71±0.76^***^	**4.62±0.69^#^**	3.91±0.19	5.28±0.32^***^	4.70±0.54
ALT (U/L)	28.25±4.99	411.57±59.30^***^	**306.43±27.82^#^**	37.70±19.85	323.58±43.15^***^	**272.36±16.73^#^**
AST (U/L)	124.35±44.45	466.68±81.96^***^	**342.23±43.18^#^**	150.05±19.87	308.15±32.93^***^	253.98±36.43
LDL (mmol/L)	0.60±0.05	0.12±0.03^***^	**0.17±0.02^#^**	0.50±0.13	0.17±0.03^***^	0.19±0.03
MDA (μmol/g protein)	0.52±0.11	1.96±0.20^***^	1.22±0.57	0.48±0.04	1.43±0.14^***^	1.19±0.19
TG (mmol/g protein)	0.21±0.05	0.34±0.03^***^	0.27±0.06	0.22±0.05	0.33±0.06^*^	0.29±0.02
TC (mmol/g protein)	27.28±8.48	55.54±3.04^***^	35.09±18.11	22.80±10.34	42.03±2.78^**^	34.73±3.36

**Note:** MCS, the mice were fed with methionine- and cystine supplement diet; MCD, the mice were fed with methionine-choline deficient diet; MCD+Silibinin, the mice were coadministrated with 100mg/kg/d silibinin by intragastric during MCD feed; **Abbreviations:** ALT, alanine transaminase; AST, aspartate aminotransferase; LDL, low-density lipoprotein; MDA, malondialdehyde; TG, triglyceride; TC, total cholesterol. * *P*<0.05; ** *P*<0.01; ** *P*<0.001; Statistic analysis were performed between MCS and MCD groups. # *P*<0.05; Statistic analysis were performed between MCD and MCD+Silibinin groups.

## Abbreviations

Lrp6: Low-density lipoprotein receptor-related protein 6
NAFLD: Nonalcoholic fatty liver disease
NASH: Nonalcoholic steatohepatitis
ALD: Alcoholic liver disease
SNPs: Single nucleotide polymorphisms
MCD: Methionine-choline deficient
MCS: Methionine- and cystine supplement
ALT: Alanine transaminase
AST: Aspartate aminotransferase
MDA: Malondialdehyde
TG: Triglyceride
TC: Total cholesterol
PNPLA3: Patatin-like phospholipase domain-containing-3
Cyp2e1: Cytochrome P450 2e1
ROS: Reactive oxygen species
