# Supplementary material for: Lrp6 Genotype affects Individual Susceptibility to Nonalcoholic Fatty Liver Disease and Silibinin Therapeutic Response via Wnt/β-catenin-Cyp2e1 Signaling
Source: Int J Biol Sci. 2021 Sep 21;17(14):3936–53. doi: 10.7150/ijbs.63732 (PMC8495406; doi:10.7150/ijbs.63732)
Supplement: Supplementary file 1 — Supplementary figures and table. [file ijbsv17p3936s1.pdf]

Additional file

Lrp6 Genotype Affects Individual Susceptibility to Nonalcoholic Fatty Liver Disease and Silibinin Therapeutic Response via Wnt/β-catenin-Cyp2e1 Signaling

Li-jie Chen[1,2,3,4], Xiu-Xian Lin[1, 2,3,4], Jing Guo[1,2,3,4], Ying Xu[1,2,3,4], Song-Xia Zhang[1,2,3,4], Dan Chen[1,2,3,4], Qing Zhao[1,2,3,4], Jian Xiao[5], Guang-Hui Lian[6], Shi-Fang Peng[7], Dong Guo[8], Hong Yang[8], Yan Shu[8], Hong-Hao Zhou[1,2,3,4], Wei Zhang[1,2,3,4], **Yao Chen** [1,2,3,4*]

1. Department of Clinical Pharmacology, Xiangya Hospital, Central South University, Changsha 410008, Hunan, China.

2. Institute of Clinical Pharmacology, Central South University, Changsha 410078, Hunan, China.

3. Engineering Research Center of Applied Technology of Pharmacogenomics, Ministry of Education, 110 Xiangya Road, Changsha 410078, P. R. China.

4. National Clinical Research Center for Geriatric Disorders, 87 Xiangya Road, Changsha 410008, Hunan, P.R. China.

5. Department of Pharmacy, Xiangya Hospital, Central South University, Changsha 410008, Hunan, China.

6. Department of gastroenterology, Xiangya Hospital, Central South University, Changsha 410008, Hunan, China.

7. Department of Hepatology and Infectious Diseases, Xiangya Hospital, Central South University, Changsha 410008, Hunan, China.

8. Department of Pharmaceutical Sciences, School of Pharmacy, University of Maryland, Baltimore, Maryland 21201. USA

*Corresponding author: Associate Prof. Yao Chen, Department of Clinical Pharmacology, Xiangya Hospital, Central South University, Changsha, 410008, P.R. China. Phone: +86-731-8480-5380, Email: cbohua@csu.edu.cn

Additional file 1.

Table S1. Comparison of odd ratios for NAFLD between different SNP genotypes and their possible influences

Gene name	SNP	Nucleotide change	Protein change	Compared genotype groups	Odd ratios for NAFLD (95% CI)	P value	Possible influence
PEMT	rs7946	g.17409560C>T	p.Val212Met	CT+TT vs CC	0.899(0.549-1.472)	0.672	Lipid import/synthesis
				TT vs CT+CC	0.733(0.143-3.757)	0.710	
PGC1β	rs7732671	g.149212243G>C	p.Ala203Pro	CG+CC vs GG	1.699(0.768-3.758)	0.191	
				GC vs GG	1.551(0.892-2.696)	0.120	
SLC27A5	rs35350976	g.59023174A>G	p.Met50Thr	AG+GG vs AA	0.894(0.399-2.006)	0.787	
				GG vs AG+AA	1.429(0.062-32.823)	0.823	
PNPLA3	rs738409	g.44324727C>G	p.Ile148Met	CG+GG vs CC	1.080(0.681-1.713)	0.744	Lipid export/oxidation
				GG vs CG+CC	1.155(0.621-2.148)	0.648	
MTTP	rs2306985	g.100516022C>G	p.His297Gln	CG+GG vs CC	0.930(0.468-1.848)	0.836	
				GG vs CG+CC	1.070(0.680-1.682)	0.770	
CYP2E1	rs6413419	g.135345675G>T	p.Val179Phe	Only GG genotype	-	-	Lipid peroxidation
L-FABP	rs2241883	g.88424066T>C	p.Thr94Ala	**CT+CC vs TT**	**0.606(0.380-0.968)**	**0.036**	**Cholesterol absorption/synthesis**
				CC vs CT+TT	0.955(0.304-3.000)	0.937	
FASN	rs2228305	g.80042792C>T	p.Val1483Ile	CT+TT *vs* CC	1.470(0.125-17.291)	0.760	Fatty acid/triglyceride synthesis
				CT vs CC	0.150(0.012-1.834)	0.138	
ApoE	rs440446	g.45409167C>G	p.Asn14Lys	CG+GG vs CC	0.925(0.574-1.490)	0.749	VLDL synthesis/export
				GG vs CG+CC	1.067(0.570-1.996)	0.839	
mTOR	rs28990992	g.11249789G>C	p.Glu51Asp	CG+CC vs GG	0.878(0.377-2.043)	0.762	
				CC vs CG+GG	0.550(0.040-7.529)	0.654	
TCF7L2	rs77961654	g.114925369C>A	p.Pro200Thr	CA+AA vs CC	0.948(0.603-1.493)	0.819	Glucose metabolism/insulin resistance
				AA vs CA+CC	0.895(0.421-1.904)	0.773	
PPARG	rs1801282	g.12393125C>G	p.Pro12Ala	GG vs GC	1.271(0.687-2.351)	0.445	
IL6	rs2069849	g.22771156C>T	p.Phe201Leu	CT vs CC	2.724(0.197-37.696)	0.455	Cytokines
Fas	rs3218619	g.90762801G>A	p.Ala16Thr	Only GG genotype	-	-	Metabolic Stress

TLR4	rs4986790	g.120475302A>G	p.Asp299Gly	Only AA genotype	-	-	Steatohepatitis-endotoxin response
LRP6	rs2302685	g.12301898C>T	p.Val1062Ile	CT+TT vs CC	0.367(0.035-3.831)	0.402	**Wnt/β-catenin signaling pathway**
				**TT vs CT+CC**	**2.853(1.378-5.910)**	**0.005**	
APC1	rs459552	g.112176756T>A	p.Val1822Asp	AT+AA vs TT	1.931(0.235-15.894)	0.541	
				AA vs AT+TT	0.692(0.392-1.219)	0.202	
DVL1	rs61735963	g.1277183C>T	p.Ala157Thr	Only CC genotype	-	-	
SIRT3	rs28365927	g.236091G>A	p.Arg80Trp	GA+AA vs GG	1.557(0.927-2.616)	0.094	Apoptosis signaling pathway
				AA vs GA+GG	0.972(0.150-6.310)	0.976	
HIF3A	rs3764609	g.46823702A>G	p.Gln274Arg	AG+GG vs AA	1.186(0.742-1.895)	0.476	HIF signaling pathway
				GG vs AG+AA	1.173(0.639-2.155)	0.607	
NFKBID	rs8113704	g.36387881A>G	p.Val181Ala	GA+GG vs AA	1.062(0.618-1.826)	0.828	NFKB signaling pathway
				GG vs GA+AA	2.903(0.399-21.121)	0.292	
STAT2	rs2066811	g.56742997T>C	p.Ile464Val	only TT genotype	-	-	JAK-STAT signaling pathway

Note: "-" indicates that statistic analysis cannot be performed due to only one genotype group.

Abbreviations: *PEMT*, phosphatidylethanolamine N-methyltransferase; *PGC 1β*, peroxisome proliferator-activated receptor gamma coactivator 1β; *SLC27A5*, solute carrier family 27 member 5; *PNPLA3*, patatin-like phospholipase domain-containing protein 3; *MTTP*, microsomal triglyceride transfer protein; *CYP2E1*, cytochrome P450, family 2, subfamily E, polypeptide 1; *FABP1*, fatty acid binding protein 1; *FASN*, fatty acid synthase; *ApoE*, apolipoprotein E; *mTOR*, mechanistic target of rapamycin; *TCF7L2*, transcription factor 7 like 2; *PPARG*, peroxisome proliferator activated receptor gamma; *IL6*, interleukin 6; *Fas*, Fas cell surface death receptor; *TLR4*, toll-like receptor 4; *LRP6*, low density lipoprotein receptor-related protein 6; *APC1*, adenomatous polyposis coli; *DVL1*, dishevelled segment polarity protein 1; *SIRT3*, sirtuin3; *HIF3A*, hypoxia-inducible factor 3-alpha; *NFKBID*, NF-Kappa-B Inhibitor Delta; *STAT2*, signal transducers and activators of transcription 2.

Additional file 2.

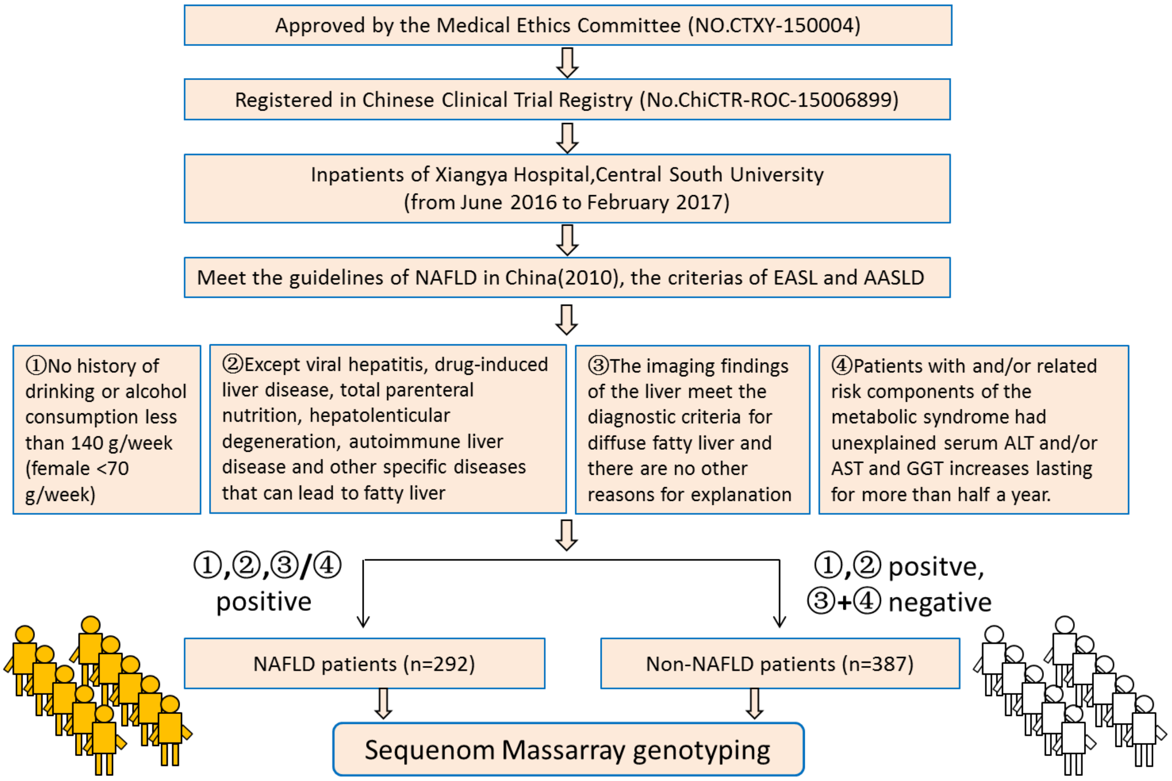

Figure S1. Schematic diagram of screening procedures for NAFLD and non-NAFLD subjects in clinical patient blood sample and data collection.

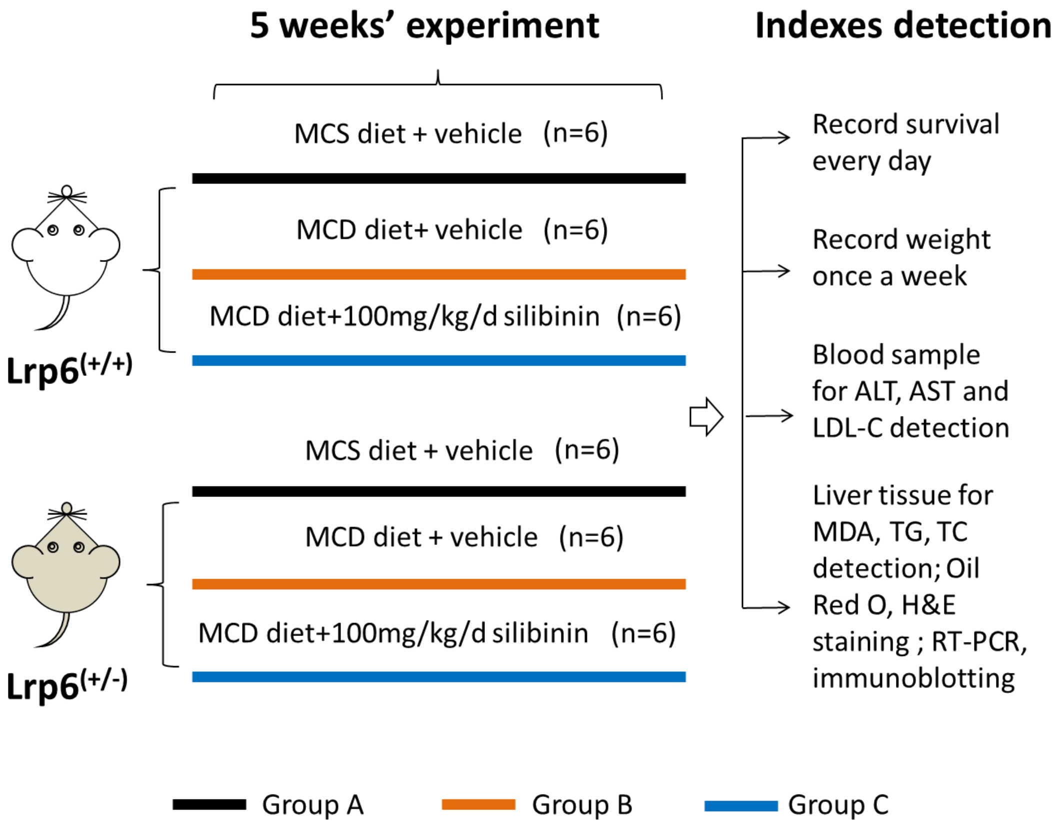

Figure S2. The experiment procedure and index detection during MCD diet-induced NAFLD and silibinin treatment between *Lrp6*$^{(+/+)}$ and *Lrp6*$^{(+/-)}$ mice, with a MCS diet as the control.

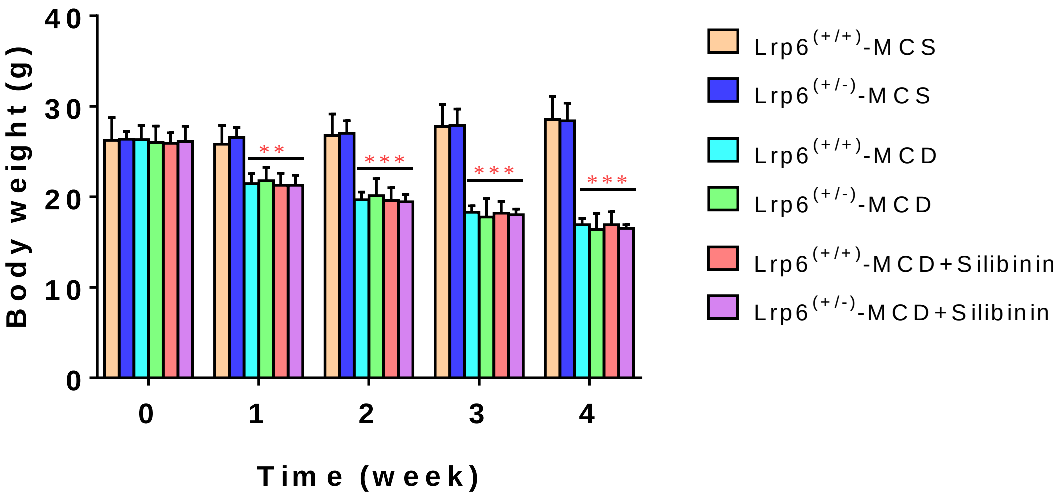

Figure S3. The changes in the mice's body weight during NAFLD moulding with and without the treatment of silibinin between the *Lrp6*$^{(+/+)}$ and the *Lrp6*$^{(+/-)}$ mice groups.

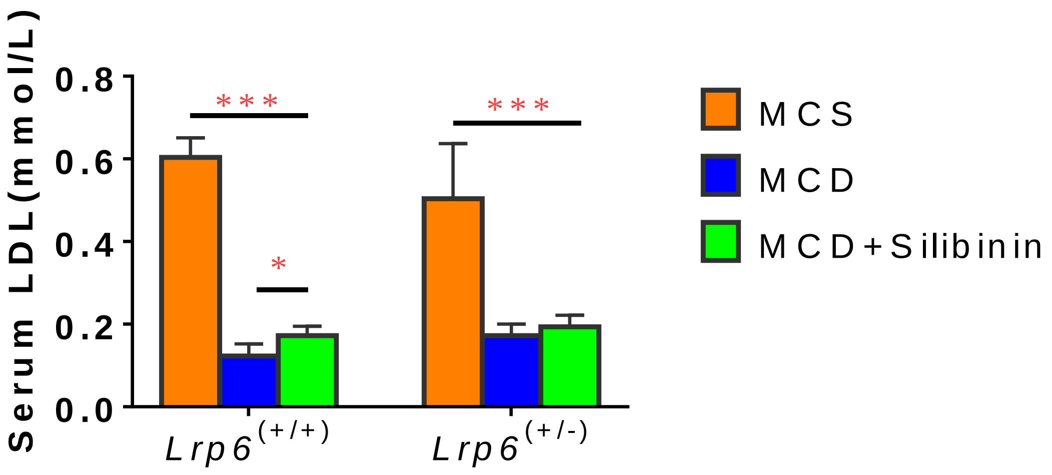

Figure S4. Changes in the LDL level during NAFLD moulding with and without the treatment of silibinin between the *Lrp6*$^{(+/+)}$ and the *Lrp6*$^{(+/-)}$ mice groups.

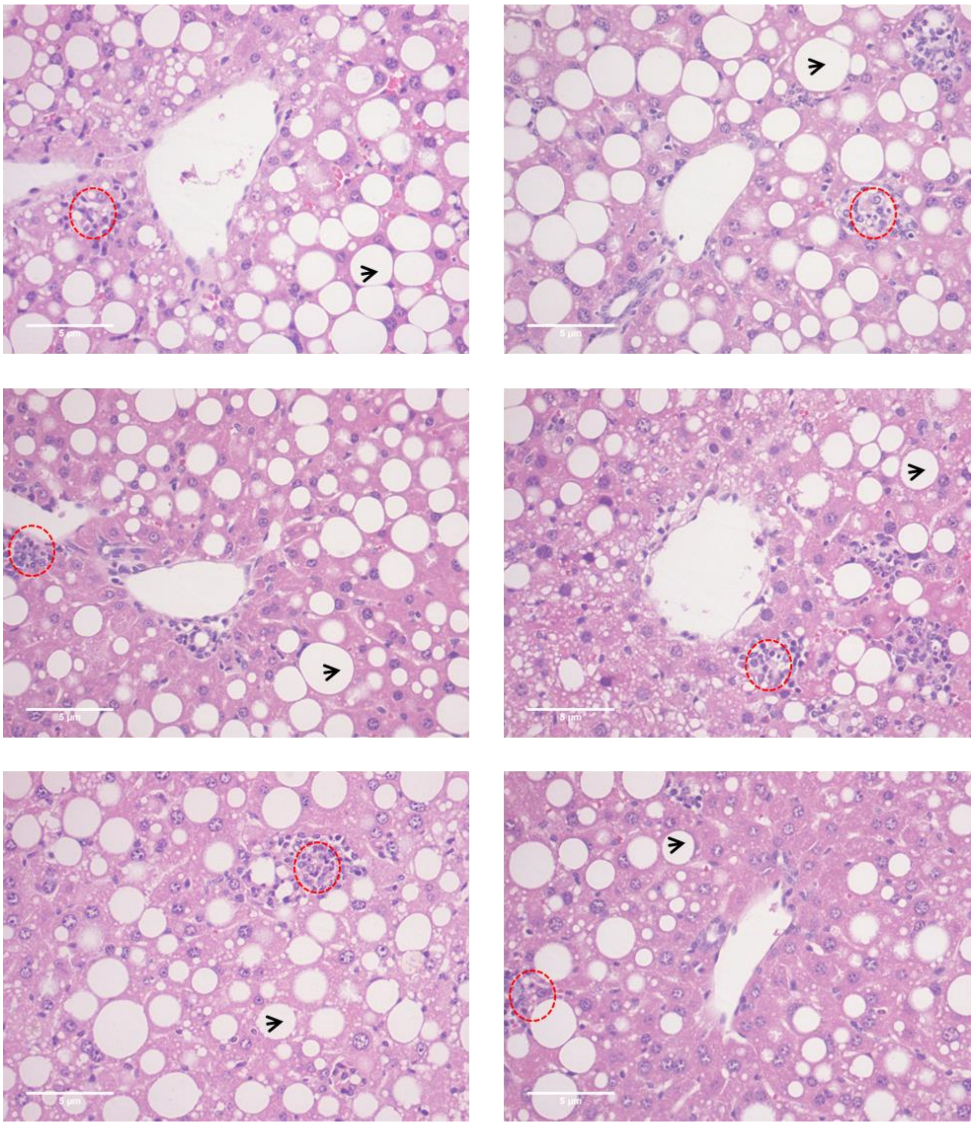

Figure S5. Additional H&E figures for inflammatory and fibrotic phenotypes of MCD-induced NAFLD mice model. Macrovesicular steatosis indicated with arrow and necroinflammatory foci indicated with circular broken lines (Scale bar, 5μm).
